# Prognostic value of procalcitonin in patients after elective cardiac surgery: a prospective cohort study

**DOI:** 10.1186/s13613-016-0215-8

**Published:** 2016-11-23

**Authors:** Matthias Klingele, Hagen Bomberg, Simone Schuster, Hans-Joachim Schäfers, Heinrich Volker Groesdonk

**Affiliations:** 1 Division of Nephrology and Hypertension, Department of Medicine, Saarland University Medical Center, University of Saarland, Homburg/Saar, Germany; 2 Department of Medicine, Hochtaunuskliniken, Usingen, Germany; 3 Department of Anesthesiology, Intensive Care Medicine and Pain Medicine, Saarland University Medical Center, University of Saarland, Kirrbergerstrasse, 66421 Homburg/Saar, Germany; 4 Department of Thoracic and Cardiovascular Surgery, Saarland University Medical Center, University of Saarland, Homburg/Saar, Germany

**Keywords:** Cardiac surgery, Procalcitonin, Risk factor, Delayed complications

## Abstract

**Background:**

Procalcitonin (PCT) is a well-known prognostic marker after elective cardiac surgery. However, the impact of elevated PCT in patients with an initially uneventful postoperative course is still unclear. The aim of this study was to evaluate PCT levels as a prognostic tool for delayed complications after elective cardiac surgery.

**Methods:**

A prospective study was performed in 751 patients with an apparently uneventful postoperative course within the first 24 h after elective cardiac surgery. Serum PCT concentration was taken the morning after surgery. All patients were screened for the occurrence of delayed complications. Delayed complications were defined by in-hospital death, intensive care unit readmission, or prolonged length of hospital stay (>12 days). Odds ratios (OR) [with 95% confidence interval (CI)] were calculated by logistic regression analyses and adjusted for confounders. Predictive capacity of PCT for delayed complications was calculated by ROC analyses. The cutoff value of PCT was derived from the Youden Index calculation.

**Results:**

Among 751 patients with an initially uneventful postoperative course, 117 patients developed delayed complications. Serum PCT levels the first postoperative day were significantly higher in these 117 patients (8.9 ng/ml) compared to the remaining 634 (0.9 ng/ml; *p* < 0.001). ROC analyses showed that PCT had a high accuracy to predict delayed complications (optimal cutoff value of 2.95 ng/ml, AUC of 0.90, sensitivity 73% and specificity 97%). Patients with PCT levels above 2.95 ng/ml the first postoperative day had a highly increased risk of delayed complications (adjusted OR, 110.2; 95% CI 51.5–235.5; *p* < 0.001).

**Conclusions:**

A single measurement of PCT seems to be a useful tool to identify patients at risk of delayed complications despite an initially uneventful postoperative course.

## Background

Procalcitonin (PCT) is a well-known marker after elective cardiac surgery [1–7]. It is a 116-amino acid peptide secreted from thyroid parafollicular cells as the precursor of calcitonin [8]. Regarding inflammatory response, PCT is synthesized in nearly all organs like liver, lung, kidney, intestine and almost all other tissues throughout the body [9, 10]. The production of PCT can be induced by endotoxin of gram-negative bacteria or by proinflammatory cytokines (e.g., IL-1 and IL-6 or TNF-α) [8].

High serum PCT has been described in patients with systemic infection [11], strongly correlating with the extent and severity of bacterial infections and in case of systemic inflammatory response [12, 13].

The use of cardiopulmonary bypass leads to various degrees of a systemic inflammatory response syndrome associated with an increase of PCT levels within the first 24 h postoperatively [7, 13, 14]. Cardiac patients with increased serum levels of PCT have been found to be related to the development of complications after surgery [1–7, 15]. However, these studies were not focused on patients with an initially uneventful postoperative course. If the initially postoperative course was uneventful, the elevated PCT levels were not included in the decision-making process for further therapy concepts. This was due to the fact that the predictive value of elevated PCT levels on the first postoperative in patients with initially uneventful postoperative course is still unknown. However, if these patients developed delayed complications with the need for ICU readmission, an extension of hospital stay with worse outcome was expected [16, 17].

In this prospective cohort study, we analyzed the predictive power of a single serum procalcitonin measurement in identifying patients after elective cardiac surgery at risk of delayed complications despite an initially uneventful postoperative course. Although a single value of PCT is described to have rather a discriminative impact than prognostic significance [18], in the postoperative setting, only a single measurement would be helpful.

## Methods

### Study design and setting

The study was designed as a prospective cohort study and approved by the local ethics committee (Landesärztekammer des Saarlandes; Ref. ID: 199/09). All patients scheduled for cardiac surgery between January 2010 and March 2011 at our center were screened for participation. The inclusion criterion was elective cardiac surgery with cardiopulmonary bypass, having an initially uneventful postoperative course. Exclusion criteria comprised: age <18 years, refusal to participate, planned off-pump surgery, urgent cardiac surgery, insufficient knowledge of the German language or all-cause complications in the immediate postoperative course. Written informed consent was obtained from all patients meeting the above-mentioned criteria and being included in this study. Patient demographics and perioperative data were entered into a computerized data bank in addition to the medical record chart.

### Study population

In our center, all elective patients with initially uneventful postoperative course were admitted to our intensive care unit for extubation. If the patients could be extubated within 9 h of surgery and showed an apparently uneventful postoperative course, the patients were discharged from our intensive care unit at latest the morning after surgery. The initially uneventful postoperative course is defined as: less than 24 h within an intensive care unit (ICU), the patients were in a clinically stable hemodynamic and respiratory condition, they did not show signs of stroke or bleeding necessitating surgery intervention and were without acute renal failure after the first night of surgery (defined by AKIN criteria) [19]. Delayed complications were defined as: complications occurring after initially uneventful postoperative course resulting in in-hospital death, intensive care unit readmission or the need for prolonged length of hospital stay (LOS) of more than 12 days. Medical reasons for delayed complications were mainly neurological complications, respiratory failure, cardiac arrhythmia, infections or acute kidney injury with need for dialysis. We compared patients with and without delayed complications.

### Endpoints

The primary endpoint of the study was declared as “combined adverse outcome” in which this was defined as the occurrence of any delayed complication after initially uneventful postoperative course in patients undergoing elective cardiac surgery. The secondary endpoint was all-cause mortality during the postoperative observation period.

### PCT measurement

Blood was sampled for inflammatory markers (procalcitonin, leukocytes and C-reactive protein) the day before surgery from a peripheral vein and on the first postoperative morning from a central venous catheter (8–16 h postoperatively). PCT concentration was determined in the hospital laboratory using the Elecsys BRAHMS PCT automated electrochemiluminescence assay (BRAHMS AG, Hennigsdorf, Germany) utilizing a cobas^®^ 8000 modular analyzer (Roche Diagnostics, Basel, Switzerland) according to the manufacturer’s instructions. The functional assay sensitivity (lowest quantifiable concentration with a between-run imprecision of <20%) met the Roche Diagnostics specification of 0.06 ng/ml PCT. The between-day coefficients of variation for the PCT analyses were found in Elecsys BRAHMS PCT controls with 1.75% for 0.49 ng/ml and 1.62% for 9.6 ng/ml precision.

To date, C-reactive protein and leukocytes are routinely used as the marker for infection or inflammation in our center. In contrast, PCT was not routinely determined during the study period. Therefore, PCT levels had no influence on the clinical treatment of patients. For more than 200 patients, levels of PCT were determined only subsequently after discharge.

### Statistical analysis

Continuous variables are expressed as mean ± standard deviation. Categorical variables are presented as a percentage unless otherwise stated. Chi-squared tests were performed for the comparison of frequencies between groups. For continuous variables, the differences between groups were compared using Student’s *t* tests. If values did not show normal distribution, Mann–Whitney *U* test was used. Two-sided *p* values of <0.05 were considered statistically significant.

Different receiver operating characteristic (ROC) curves were constructed to evaluate the predictive power of inflammatory markers for the occurrence of delayed complications. The Youden Index was used to calculate optimal cutoff points for inflammatory markers in prediction of delayed complications.

To determine the risk of elevated postoperative serum PCT for delayed complications, odds ratios were calculated by logistic regression analyses with 95% confidence intervals (CI). Two additional models were calculated adjusting for potential confounders. Potential confounders in model one were: preoperative age, glomerular filtration rates (eGFR, calculated by CKD-EPI formula) and EuroSCORE 2. In the second model, we further adjusted for: preoperative C-reactive protein (CRP), pulmonary hypertension, valve surgery, combination surgery, redo coronary artery bypass grafting (CABG) and/or cardiac valve surgery and cardiopulmonary bypass (CPB) time since these factors showed differences between patients developing delayed complications and those who did not. To account for possible collinearity in multiple regressions, Pearson’s or Spearman’s correlation coefficients were calculated. Variables with a positive or negative bivariate correlation exceeding +0.3 or less than −0.3 were evaluated for interactions. The goodness of fit was assessed by Hosmer–Lemeshow tests. Data analysis was performed using SPSS Statistics 19™ (IBM, Ehningen, Germany).

## Results

### Study population

**Fig. 1:**
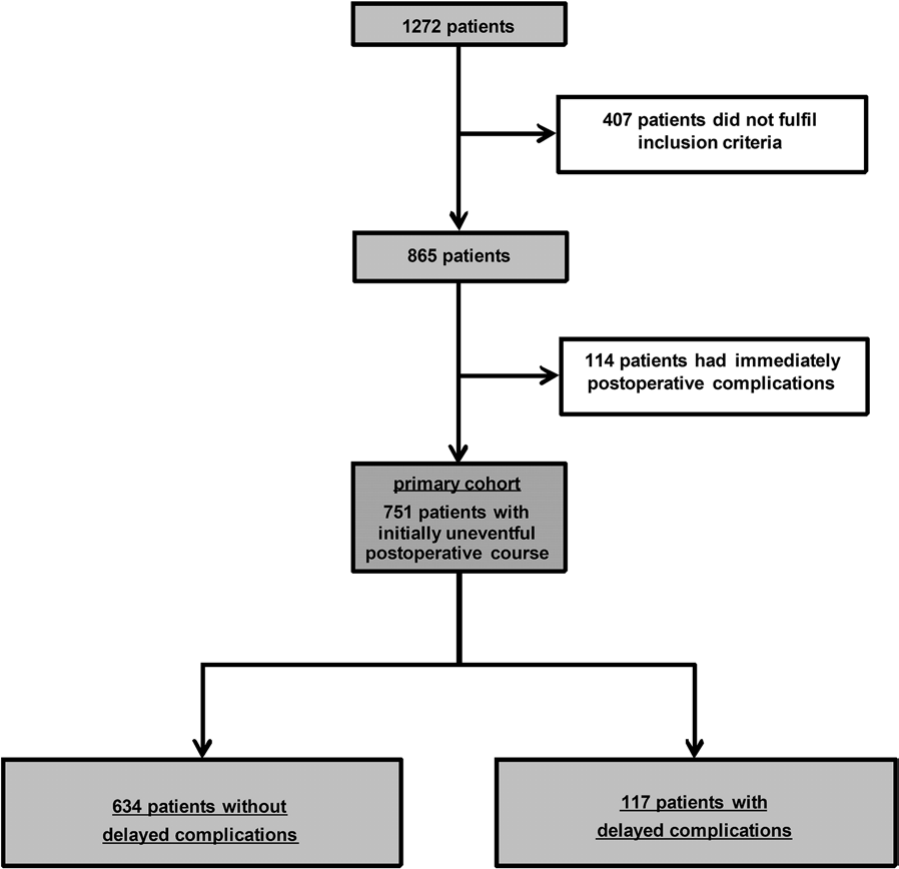
Flowchart

During the study period, 1272 patients underwent cardiac surgery with extracorporeal circulation. Of these, 407 patients did not meet inclusion criteria due to urgent or emergency surgery or refused participation. In total, 114 patients were excluded due to severe postoperative complications occurring in the immediate postoperative course. The final study population thus included 751 patients with an initially uneventful postoperative course. Of these 117 patients developed delayed complications after discharge from the intensive care unit (Fig. 1). Patients developing delayed complications were generally: older, had poorer preoperative renal function, a higher EuroSCORE 2 and lower left-ventricular ejection fraction. Operation time and CBP time were longer, and the portion of combined surgery was higher in this group. The outcome worsened in patients developing delayed complications compared without. ICU readmission was observed in 51 of 117 patients developing delayed complications. They had a significantly increased mechanical ventilation time, prolonged length of stay in the intensive care unit and hospital, as well as a higher hospital mortality (Table 1).

**Table 1:** Baseline characteristics and in-hospital outcome

Characteristic	Patients without delayed complications (*n* = 634)	Patients with delayed complications (*n* = 117)	*p* value
*Demographics*			
Gender (*n*) (%)			
Male	445 (70)	74 (65)	0.16
Age (years)	61.5 ± 15.2	67.7 ± 11.6	<*0.001*
Weight (kg)	82.6 ± 15.1	81.5 ± 81.5	0.26
Size (cm)	173 ± 9.6	171 ± 9.0	0.11
Body mass index (kg/m^2^)	27.7 ± 4.5	27.7 ± 4.9	0.76
eGFR (CKD-EPI)	86.7 ± 33.8	70.0 ± 32.4	<*0.001*
EuroSCORE 2	4.9 ± 5.6	8.0 ± 6.9	<*0.001*
Ejection fraction (%)	60.3 ± 13.9	55.4 ± 16.2	*0.009*
NYHA class	2.9 ± 0.5	2.9 ± 0.6	0.39
*Comorbidity*			
Coronary artery disease (*n*) (%)	258 (41)	57 (49)	0.13
Pulmonary hypertension (*n*) (%)	122 (19)	34 (29)	*0.019*
COPD (*n*) (%)	34 (5)	10 (9)	0.20
Status past stroke (*n*) (%)	23 (4)	4 (4)	1
Insulin-dependent diabetic (*n*) (%)	27 (4)	10 (9)	0.061
*Intraoperative*			
Procedure type (*n*) (%)			
CABG surgery	228 (36)	49 (42)	0.25
Valve surgery	442 (70)	92 (79)	0.059
Combination surgery	183 (29)	55 (47)	<*0.001*
Redo CABG and/or valve	68 (11)	12 (10)	1
Operation time (min)	159 ± 44.5	189 ± 63.8	<*0.001*
CPB time (min)	77 ± 28.6	100 ± 42.1	<*0.001*
*Outcome*			
Mech. ventilated (h) (min–max)	5.0 ± 2.1	35.4 ± 102	<*0.001*
Length of stay (days)			
In ICU (days) (min–max)	0.9 ± 0.3	3.1 ± 6.0	<*0.001*
In hospital (days) (min–max)	9.3 ± 1.6	20.1 ± 11.3	<*0.001*
Hospital mortality (%)	0 (0)	4 (3)	*0.001*

Data are expressed as mean ± standard deviation

Statistically significant *p* values are in italics (*p* < 0.05)

*eGFR (CKD-EPI)* glomerular filtration rates, calculated by CKD-EPI-formula (Chronic Kidney Disease Epidemiology Collaboration). *NYHA class* New York Heart Association functional classification, *COPD* chronic obstructive pulmonary disease, *CABG surgery* coronary artery bypass grafting surgery, *CPB time* cardiopulmonary bypass time, *ICU* intensive care unit

### Serum procalcitonin level and delayed complications

**Fig. 2:**
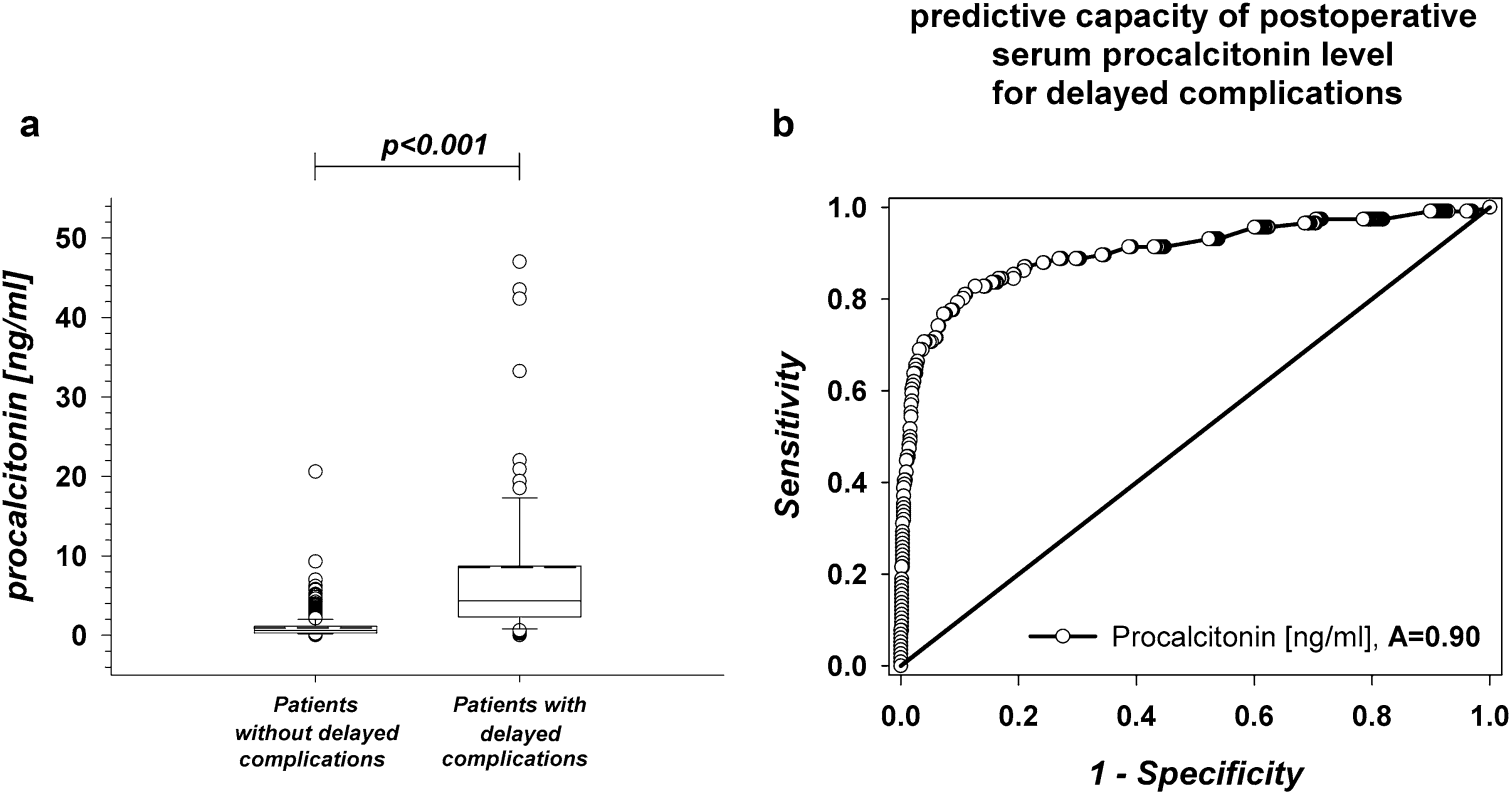
Postoperative serum levels of procalcitonin and ROC curve analysis

Comparing patients with and without delayed complications, PCT levels were significantly different the morning after surgical intervention (8.6 ± 13.5 and 0.9 ± 1.2, respectively; *p* < 0.001; Table 2; Fig. 2a).

**Table 2:** Preoperative and postoperative serum levels of C-reactive protein, leukocytes and procalcitonin (PCT)

Characteristic	Patients without delayed complications (*n* = 634)	Patients with delayed complications (*n* = 117)	*p* value
*Inflammatory markers*			
Preoperative			
PCT (ng/ml) (min–max); normal: <0.5 ng/ml	0.03 ± 0.14	0.09 ± 0.28	<*0.001*
Leukocytes (×10^9^/l) (min–max)	7.4 ± 3.9	7.8 ± 2.5	0.12
C-reactive protein (mg/l) (min–max); normal: <5 mg/l	7.4 ± 16.8	16.2 ± 33.7	*0.001*
Postoperative			
PCT [ng/ml] (min–max); normal: <0.5 ng/ml	0.9 ± 1.2	8.6 ± 13.5	<*0.001*
Leukocytes (×10^9^/l) (min–max)	11.3 ± 4.3	12.6 ± 4.2	<*0.001*
C-reactive protein (mg/l) (min–max); normal: <5 mg/l	70.0 ± 29.7	74.8 ± 33.7	0.24

Data are expressed as mean ± standard deviation

Statistically significant *p* values are in italics (*p* < 0.05)

ROC analyses of postoperative PCT serum levels and the occurrence of any delayed complication revealed an AUC of 0.90 with an optimal cutoff value of 2.95 ng/ml (sensitivity: 0.73, specificity: 0.97; Fig. 2b).

Looking in detail at the occurrence of different complications, mean PCT levels on the first postoperative day varied between 4.4 ± 4.8 and 12.8 ± 17.3 ng/ml. Thereby, the corresponding predictive capacity of PCT levels showed an AUC between 0.75 and 0.90 (Table 3).

**Table 3:** Reasons for delayed complications and predictive accuracy of procalcitonin (PCT) for these complications

(*n* = 751)	Predictive accuracy of PCT for delayed complications							
	Incidence (*n*)	PCT	AUC	95% CI	*p* value	Cutoff (ng/ml)	Sensitivity (%)	Specificity (%)
Respiratory failure	16	7.6 ± 10.1	0.90	0.85–0.95	<0.001	>3.1	81	90
Cardiac arrhythmia	40	6.0 ± 7.3	0.90	0.84–0.95	<0.001	>2.3	90	87
Other complications	21	12.8 ± 17.3	0.89	0.79–0.99	<0.001	>3.0	86	89
Infection								
Instability or sepsis	44	11.5 ± 17.8	0.89	0.82–0.95	<0.001	>2.3	86	87
SIRS/sepsis	29	4.4 ± 4.8	0.75	0.62–0.87	<0.001	>1.3	74	71

In some patients more than one complication occurred. Data are expressed as mean ± standard deviation

*AUC* area under the curve

After multivariable analysis, patients with PCT levels above the cutoff value of 2.95 ng/ml the first postoperative day had a 66 times higher risk of developing delayed complications compared to those with PCT levels below this threshold. Even after adjustment for potential confounders, a serum PCT level >2.95 ng/ml remained an independent and strong risk factor (adjusted 2 OR: 110.16, 95% CI 51.53–235.52; Table 4). Goodness of fit for each adjusted model was assessed by Hosmer–Lemeshow tests and was not statistically significant.

**Table 4:** Serum procalcitonin levels on the first postoperative day as predictor for delayed complications

	Postoperative cutoff PCT > 2.95 ng/ml and risk of delayed complications		
	Odds ratio	95% CI	*χ* ^2^ (*p* value)
Crude	66.38	36.73–119.96	265.67 (<0.001)
Adjusted 1	89.76	45.19–178.30	299.22 (<0.001)
Adjusted 2	110.16	51.53–235.52	328.79 (<0.001)

Data are expressed as odds ratios with 95% confidence intervals (CI). Potential confounders in model one (adjusted 1) were: preoperative age, glomerular filtration rates and EuroSCORE 2. In the second model (adjusted 2), we further adjusted for: preoperative C-reactive protein, pulmonary hypertension, valve surgery, combination surgery, redo coronary artery bypass grafting and/or cardiac valve surgery and cardiopulmonary bypass time

### Preoperative and postoperative parameters of inflammation

Preoperative levels of CRP and PCT were different in patients developing delayed complications and those who did not (CRP: 7.4 ± 16.8 vs. 16.2 ± 33.7, *p* = 0.001; PCT: 0.03 ± 0.14 vs. 0.09 ± 0.28, *p* < 0.001). However, leukocytes were similar between both groups (leukocytes: 7.4 ± 3.9 vs. 7.8 ± 2.5, *p* = 0.12). All patients showed postoperatively a comparable rise of CRP and leukocytes (Table 2). With respect to the postoperative CRP levels or the number of leukocytes, it was not possible to distinguish patients who would develop delayed complications and those who would not. ROC analyses showed that elevated postoperative PCT serum levels had the highest discrimination to predict delayed complication compared with CRP and leukocytes (Table 5). Postoperative PCT serum levels showed the highest positive and negative predictive value compared with all other inflammatory markers.

**Table 5:** Data are expressed as AUC (area under the curve) with 95% confidence interval (CI)

	ROC analysis to predict delayed complications (n = 751)								
	AUC	95% CI	*p* value	Cutoff	Likelihood ratio	Sensitivity (%)	Specificity (%)	PPV (%)	NPV (%)
*Preoperative*									
Procalcitonin (ng/ml)	0.58	0.52–0.64	0.007	>0.05	2.32	28	88	30	87
Leukocytes (x10^9^/l)	0.57	0.52–0.63	0.02	>9.3	1.97	27	86	27	87
C-reactive protein (mg/l)	0.60	0.54–0.65	<0.001	>11.6	2.07	27	87	28	87
*Postoperative*									
Procalcitonin (ng/ml)	0.90	0.87–0.94	<0.001	>2.95	21.83	73	97	82	95
Leukocytes (×10^9^/l)	0.62	0.56–0.67	<0.001	>13.7	1.82	35	81	25	87
C-reactive protein (mg/l)	0.53	0.48–0.59	0.24	>46.4	1.06	85	21	16	88

Different receiver operating characteristic (ROC) curves were constructed to evaluate the predictive power for delayed complications

*PPV* the positive predictive value, *NPV* the negative predictive

## Discussion

The most impressive finding of this study is that a single postoperative measurement of procalcitonin (PCT) levels predicts delayed complications in patients undergoing elective cardiac surgery despite an apparently uneventful postoperative course. The morning after surgical intervention, PCT levels were significantly increased in patients with delayed complications in univariate and multivariate analysis. ROC analyses revealed that increased PCT levels had a highly predictive accuracy to detect these delayed complications.

The use of cardiopulmonary bypass (CPB) leads to varying degrees of tissue inflammation and cytokine liberation [20] because it triggers a systemic inflammatory response syndrome with raising PCT levels during the first 24 h after surgery [13, 21]. PCT peak levels seem to be associated with different types of surgery [21]. It is well known that elevated serum PCT has been described as an independent predictor of hospital all-cause mortality within the intensive care setting [22] and correlates with poor outcome after cardiac surgery [3, 4, 6, 7, 23]. However, the meaning of elevated PCT levels in patients with an uneventful postoperative course is still unclear.

If patients with elevated PCT levels are at higher risk to develop delayed complications despite an initially uneventful postoperative course, PCT could be involved in the decision-making process for further therapy concepts. Delayed complications resulting in readmission to intensive care unit lead to prolonged hospital stay and increased mortality [16, 17]. That was also seen in our patients with delayed complications. Therefore, early identification of patients at risk of delayed complications is needed.

Cases with delayed complications mostly result in longer hospital stay. However, a variety of factors can influence LOS with significant differences existing between the health systems of different nations. The current study was conducted in Germany. Patients are normally admitted to hospital the day before surgery and generally discharged only when home situation is ensured or direct transfer to a rehabilitation facility is possible. These factors can affect discharge planning. In our center, we aim for a total LOS < 10 days. A study by Kiessling and colleagues within Germany found a mean LOS of 10.3 ± 2.5 days in patients with a successful fast track and a mean LOS of 16.5 ± 16.3 days when fast-track failure occurred [17]. We chose LOS > 12 days as cutoff value for prolonged stay as we feel that this would take into account the aforementioned factors. Moreover, in this way it would be possible to incorporate clinically relevant delayed complications accounting for a longer hospital stay.

In order to evaluate PCT as a marker to identify patients at risk of delayed complications, we selected only patients with an initially uneventful postoperative course and used logistic regression analysis adjusting for confounders. After adjustment of confounders, PCT remained an independent predictor.

ROC curve analysis of postoperative PCT levels revealed a highly predictive value for the occurrence of any delayed complications; the prediction of a specific complication regarding the various complications encountered was not possible. In line with our results, previous studies found that PCT levels revealed a highly predictive value for the occurrence of complications after cardiac surgery [3–5, 24, 25]. Nevertheless, PCT levels were not found to be specific for an explicit complication, but predict various complications such as SIRS, sepsis, infection without circulatory instability, myocardial infarction and respiratory failure [3–5, 24–26].

The use of cardiopulmonary bypass is associated with an increase of PCT and CRP levels [7, 14]. Our results confirm this postoperative increase in inflammatory markers. All three markers, PCT, CRP and leukocyte, were increased postoperatively. However, patients at risk of delayed complications could only be identified by means of elevated PCT levels. The failure of CRP as a prognostic marker after cardiac surgery is in line with previous studies [5, 14]. White cell count showed a significant difference comparing patients with delayed complications and all remaining patients. However, in clinical practice this small difference is not considered as “different.”

Postoperative delayed complications would be dependent on many factors. However, this raises a crucial question: What is the reason for different increase of PCT in patients? As shown in Table 1, comorbidity and intraoperative parameters were different and could explain more postoperative complications. However, after surgery the patients showed no clinical signs of complications. Nevertheless, it is known that increased PCT levels are associated with many inflammatory diseases (e.g., blood stream infection, pneumonia, renal failure or heart failure) [11, 27–30], all known to possibly occur after cardiac surgery. It can be summarized that it seems that increased PCT levels are associated with worse global health condition. We hypothesize that the raise of PCT after surgery is an early warning signal of upcoming complications associated with proinflammatory states.

Regarding our results, monitoring postoperative PCT levels could be a helpful tool to predict likelihood of delayed complications independently of the patient’s clinical appearance the first postoperative day. That can be easily integrated into clinical practice and help decision-making processes for planning postoperative monitoring. We assume that the focus to see every patient and therapy more individually could be helpful to improve patient outcome.

## Limitations

Our major limitation is that we built our models from a single population. Due to the study design, physicians and caregivers were not blinded to any extent during this prospective observational study. However, levels of PCT had no influence on the clinical treatment of patients as PCT was not routinely determined during the study period.

Another limitation of the current study is that the decision rule and the evaluation of the performance of this rule were both determined from the same population of patients. In particular, the sensitivity and specificity of a procalcitonin cutoff value for delayed complications need to be validated in a separate population of patients. Moreover, patients developing delayed complications showed higher preoperative CRP and PCT levels with a higher risk of adverse outcome before surgery. However, there was only a low discrimination to predict delayed complication after surgery. Although both a large number of patients and several different surgical procedures are included in the current study, confirmation of the above results is suggested as part of a multicenter study.

## Conclusions

In this single-center, observational cohort study, a single measurement of serum PCT level the morning after elective cardiac surgery seems to be an independent predictor for delayed complications in patients with an apparently uneventful postoperative course. Detection of such at-risk patients prior to ICU discharge could probably avoid readmission and ensure adequate monitoring, enabling rapid treatment of emerging complications. In conclusion, we think, by monitoring postoperatively, PCT is in certain circumstances an interesting concept in which the true clinical value of this idea should be evaluated in a large prospective study.

## Abbreviations

CBP: cardiopulmonary bypass
CRP: c-reactive protein
ICU: intensive care unit
PCT: procalcitonin
